# Sepsis chez l’enfant: protocole d’orientation rapide vers la réanimation pédiatrique

**DOI:** 10.11604/pamj.2021.39.189.28917

**Published:** 2021-07-08

**Authors:** Ouissal Aissaoui, Meryem El-bouz, Ahmed Aziz Bousfiha, Widad Gueddari, Abdelaziz Chlilek

**Affiliations:** 1 Laboratoire d´Immunologie Clinique, Inflammation et Allergie (LICIA), Faculté de Médecine et de Pharmacie de Casablanca, Université Hassan II de Casablanca, Casablanca, Maroc,; 2 Service d´Accueil des Urgences Pédiatriques, Hôpital Mère-Enfant Abderrahim Harouchi, Centre Hospitalier Universitaire Ibn Rochd de Casablanca, Faculté de Médecine et de Pharmacie de Casablanca, Université Hassan II de Casablanca, Casablanca, Maroc,; 3 Service de Pédiatrie 1, Hôpital Mère-Enfant Abderrahim Harouchi, Centre Hospitalier Universitaire Ibn Rochd de Casablanca, Laboratoire d´Immunologie Clinique, Inflammation et allergie (LICIA) Faculté de Médecine et de Pharmacie de Casablanca, Université Hassan II de Casablanca, Casablanca, Maroc

**Keywords:** Sepsis, enfant, réanimation pédiatrique

## Aux éditeurs du Pan Africa Medical journal

Le sepsis chez l´enfant est une urgence diagnostique et thérapeutique. Il s´agit d´une entité sous diagnostiquée et le retard de prise en charge est grevé d´une mortalité élevée. Cette dernière dépasse les 10% en cas de sepsis et les 40% en cas de choc septique [1]. La mortalité imputable au sepsis et au choc septique chez l´enfant est encore particulièrement élevée dans les pays en voie de développement. Nous proposons, à travers cette lettre, un protocole d´orientation vers la réanimation pédiatrique appliqué à l´enfant admis pour suspicion de sepsis. Ce protocole permettrait d´unifier le langage entre pédiatres, urgentistes pédiatres et réanimateurs pédiatres et par conséquent, une reconnaissance précoce, une évaluation adéquate des défaillances ainsi qu´une orientation rapide vers la réanimation pédiatrique. Ainsi, pour définir le sepsis et le choc septique chez l´enfant, nous avons adopté les recommandations de la 3^e^ conférence de consensus sur le sepsis ou « Sepsis 3 » validées chez l´adulte [1] puis adaptées à l´enfant en 2017 [2] et ce, en attendant la réactualisation des définitions pédiatriques par le groupe de travail sur la définition du sepsis chez l´enfant de la « Society of Critical Care Medicine » [3]. Le sepsis étant défini, chez l´enfant comme chez l´adulte, par une dysfonction d´organe menaçant le pronostic vital et secondaire à une dérégulation de la réponse immunitaire de l´hôte à l´infection. Il est associé à une mortalité qui dépasse les 10%. Sur le plan pratique, il s´agit d´un enfant admis pour infection et qui a un score SOFA pédiatrique (Tableau 1) supérieur ou égal à 2.

**Tableau 1:** score sequential sepsis-related organ failure assessment (SOFA) pédiatrique traduit et adapté

	0	1	2	3	4
Neurologique: Score de Glasgow	15	13-14	10-12	6-9	<6
Respiratoire: PaO2/FiO2 Ou SpO2/FiO2	≥ 400	300-399	200-299	100-199	< 100
	≥ 292	264-291	221-264	148-220	<148
Hémodynamique PAM en mmHg					
< 1 mois	≥ 46	< 46	Drogues en μg/Kg/min: Dopamine ≤ 5 Ou Dobutamine	Drogues en μg/Kg/min: Dopamine 5-15 Ou Noradré* ≤ 0,1 Ou Adré** ≤ 0,1	Drogues en μg/Kg/min: Dopamine >15 Ou Noradré* > 0,1 Ou Adré** > 0,1
[1 mois - 1 ans[	≥ 55	< 55			
[1 an - 2 ans[	≥ 60	< 60			
[2 ans - 5 ans[	≥ 62	< 62			
[5 ans - 12 ans[	≥ 65	< 65			
[12 ans - 18 ans[	≥ 67	< 67			
≥ 18 ans	≥ 70	< 70			
Coagulation: Plaquettes /μL	≥ 150 000	100 000-149 000	50 000-99 000	20 000-49 000	<20 000
Hépatique: Bilirubine mg/dL	< 1,2	1,2 - 1,9	2,0 - 5,9	6,0 - 11,9	> 12,0
Rénal: Créatinine en mg/l					
< 1 mois	< 8	8 - 9	10 - 11	12 - 15	≥ 16
[1 mois - 1 ans[	< 3	3 - 4	5 - 7	8 - 11	≥ 12
[1 an - 2 ans[	< 4	4 - 5	6 - 10	11 - 14	≥ 15
[2 ans - 5 ans[	< 6	6 - 8	9 - 15	16 - 12	≥ 23
[5 ans - 12 ans[	< 7	7 - 10	11 - 17	18 - 25	≥ 26
[12 ans - 18 ans[	<10	10 - 16	17 - 28	29 - 41	≥ 42
≥ 18 ans	<12	12 - 19	20 - 34	35 - 49	≥ 50

* : Noradrénaline ** : Adrénaline

Le choc septique est un sous-groupe de sepsis au cours duquel les dysfonctions circulatoires, cellulaires et métaboliques sont sévères et associées à une mortalité élevée, comparée à celle liée au sepsis seul. Cette dernière dépasse les 40%. Sur le plan pratique, il s´agit d´un enfant qui répond aux critères du sepsis et chez qui l´hypotension artérielle persiste malgré un remplissage adéquat (≥ 40ml/Kg) et le taux de lactates est supérieur ou égal à 2 mmol/l. Il nécessite le recours aux vasopresseurs pour maintenir une pression artérielle moyenne (PAM) correcte par rapport à l´âge. Les objectifs de PAM en fonction de l´âge sont présentés comme suit: nouveau-né - 1 mois: ≥46mmHg; [1 mois - 1 an [ : ≥55mmHg; [1 an - 2 ans [ : ≥60mmHg; [2 ans - 5 ans [: ≥62mmHg; [5 ans - 12ans [ : ≥65mmHg; [12 ans - 18 ans [ : ≥67mmHg.

Concernant les scores de gravité, nous avons adopté le score SOFA pédiatrique que nous avons traduit et modifié afin de nous adapter aux valeurs biologiques fournies par notre laboratoire. Le score « SOFA » désigne « Sequential sepsis-related Organ Failure assessment ». Il vise à évaluer les dysfonctions d´organe relatives au sepsis. Il a été validé chez l´adulte depuis la réactualisation de la campagne de survie au sepsis en 2016. Le SOFA pédiatrique est une adaptation du score « SOFA » adulte qui a été validé en pédiatrie [2,4]. Il évalue 6 défaillances: neurologique: reposant sur l´évaluation du score de Glasgow; respiratoire: reposant sur le calcul du rapport PaO_2_/FiO_2_, ou à défaut du rapport SpO_2_/FiO_2_. (PaO_2_: pression artérielle en oxygène; FiO_2_: fraction inspirée en oxygène). Etant conscients des difficultés relatives à la réalisation de gazométrie artérielle dans le cadre des urgences pédiatriques des pays en voie de développement, nous proposons d´adopter le rapport SpO_2_/FiO_2_; hémodynamique: reposant sur l´évaluation de la PAM en fonction de l´âge et du recours aux drogues vasoactives; hématologique : reposant sur l´évaluation du taux de plaquettes; hépatique: reposant sur l´évaluation du taux de la bilirubine; rénale: reposant sur l´évaluation de la créatininémie en fonction de l´âge.

Le score SOFA pédiatrique est présenté sur le Tableau 1. Ainsi, nous proposons que tout enfant ayant un score Sofa pédiatrique ≥ à 2 soit orienté vers la réanimation pédiatrique. La prise en charge initiale de l´enfant en sepsis que nous proposons émane des recommandations de la compagne de survie au sepsis consacrées à l´enfant et publiées en Février 2020 [5]. Cette dernière repose sur 6 points essentiels: assurer un abord vasculaire par la prise d´une voie veineuse périphérique ou d´une voie intra-osseuse; réaliser une Hémoculture et par la même occasion, prélever un tube EDTA (ethylenediaminetetraacetic acid) pour la numération formule sanguine et un tube sec pour doser la créatinine et la bilirubine (pour calculer le score SOFA pédiatrique), ainsi que la protéine C réactive (CRP) et le bilan hydro-électrolytique; démarrer une antibiothérapie à base de Ceftriaxone 75 mg/Kg/j, un aminoside est rajouté en fonction de l´orientation clinique et de l´absence de contre-indications doser les lactates (en fonction de la disponibilité); remplir par un bolus de sérum salé 0,9% à raison de 20ml/Kg, à répéter une à deux fois sans dépasser 60 ml/Kg durant la première heure; administrer les vasopresseurs à base de noradrénaline en première intention, en cas de non réponse au remplissage précédent. Pour cela, la voie veineuse périphérique peut être utilisée, en attendant la prise d´une voie veineuse centrale.

## Conclusion

L´adoption de protocoles simples et adaptés aux pays en voie de développement permettrait d´améliorer la prise en charge et de diminuer la mortalité liée au sepsis et choc septique de l´enfant.
